# Optical Methods Based on Ultraviolet, Visible, and Near-Infrared Spectra to Estimate Fat and Protein in Raw Milk: A Review

**DOI:** 10.3390/s20123356

**Published:** 2020-06-13

**Authors:** Abraham Gastélum-Barrios, Genaro M. Soto-Zarazúa, Axel Escamilla-García, Manuel Toledano-Ayala, Gonzalo Macías-Bobadilla, Daniel Jauregui-Vazquez

**Affiliations:** 1 Facultad de Ingeniería Campus Amazcala, Universidad Autónoma de Querétaro, Carr. Chichimequillas S/N Km 1, Amazcala, El Marqués 76265, Mexico; aescamilla514@alumnos.uaq.mx (A.E.-G.); toledano@uaq.mx (M.T.-A.); gonzalo.macias@uaq.mx (G.M.-B.); 2 Departamento de Electrónica, División de Ingenierías, Campus Irapuato-Salamanca, Universidad de Guanajuato, Carretera Salamanca-Valle de Santiago Km. 3.5+1.8 Comunidad de Palo Blanco, Salamanca, Guanajuato 36787, Mexico; jaureguid@ugto.mx

**Keywords:** fiber optic sensor, spectroscopy, raw milk, milk quality, light scattering

## Abstract

The present manuscript focuses on reviewing the optical techniques proposed to monitor milk quality in dairy farms to increase productivity and reduce costs. As is well known, the quality is linked to the fat and protein concentration; in addition, this issue is crucial to maintaining a healthy herd and preventing illnesses such as mastitis and ketosis. Usually, the quality of the milk is carried out with invasive methods employing chemical reagents that increase the time analysis. As a solution, several spectroscopy optical methods have been proposed, here, the benefits such as non-invasive measurement, online implementation, rapid estimation, and cost-effective execution. The most attractive optical methods to estimate fat and protein in cow’s milk are compared and discussed considering their performance. The analysis is divided considering the wavelength operation (ultraviolet, visible, and infrared). Moreover, the weaknesses and strengths of the methods are fully analyzed. Finally, we provide the trends and a recent technique based on spectroscopy in the visible wavelength.

## 1. Introduction

In the dairy industry, two of the main parameters that give information about quality is fat and protein. Its content determines the price of the product, the health of the herd, prevent illnesses, and gives quality control references [1]. The best way to monitor cow health is performing a regular analysis of the produced milk, and evaluating the ratio between fat and protein, preventing problems such as mastitis, ketosis, ruminal acidosis, among others [2,3].

The most-used method to determine fat is the Gerber laboratory technique and Lowry method for protein; besides its sensitiveness, a large amount of time is needed to perform assays. It is also susceptible to the interference caused by compounds used in the preparation of the samples [4], chemical reagents are needed that, in many cases, are harmful for the laboratory people that manage them.

The commercial milk analyzers have been used for worldwide milk-processing corporations to estimate the milk quality; the most accepted in practice are the MilkoScan (Foss Electric A/S, Hillerød, Denmark) and the Lactoscan (Milkotronic Ltd., Nova Zagora, Bulgaria). Despite its common use, both systems are offline analyzers. The MilkoScan is based on the Fourier Transform Infrared (FTIR) analysis of the wavelength region between 2500 and 25,000 nm. On the other hand, the Lactoscan determination is based on the ultrasonic technique, measuring the time that the sound crosses the sample of milk. In both, the milk samples must be transported to the laboratory where the system is installed, then, after a few seconds, the results are shown. Finally, the milk sample is disposed. As a consequence, several research groups have been developed many techniques in last decades, where the principles of operation are based on nuclear magnetic resonance (NMR); conventional digital imaging; ultraviolet (UV) illumination, visible (VIS) light effects, and infrared (IR) spectroscopy; electrical conductivity; fiber-optic sensors; among others.

In this review, the optical techniques for monitoring fat and protein content in raw milk are presented, with the objective of developing a cheap, online, and non-invasive system. The principal results from each technique are reviewed according to UV, VIS, and IR wavelength range. Finally, a trend in the field of optical techniques to estimate fat and protein content in raw milk is presented, as well as the current method that the research team is developing.

## 2. Optical Properties of Milk

**Figure 1:**
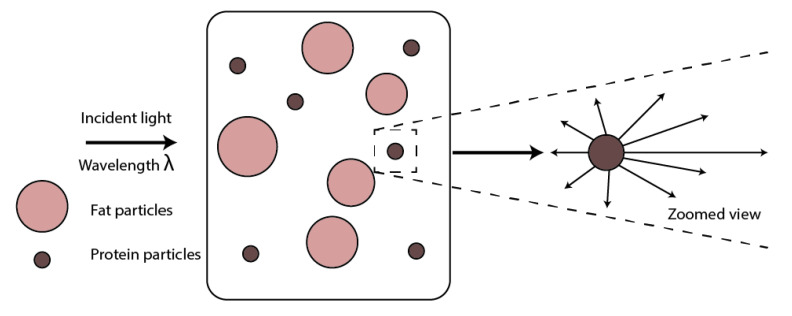
Scatter light effects generated by fat and protein particles. The incident wavelength is smaller than the diameter of both particles. In the zoomed view, the anisotropic Mie scattering is represented.

As is well known, the milk is a complex liquid composed of water (87%), fat (3.7%), protein (3.4%), lactose (4.8%) and other elements (0.7%), such as acid citric and minerals [5]. Then, the light–milk interaction will be governed by the relationship between the size of particles suspended and the wavelength of incident light [3,6,7,8]. Considering that the lipids have a diameter between 0.1 to 10 µm [7,9], and a casein protein has a diameter ranging from 50 to 680 nm [9], the milk–light interaction will be described by the Lorenz–Mie theory [10]. This considers the molecules involved as a spherical particle and the wavelength of incident light as smaller than the size of the molecules; this relationship is described by 0.1 λ<P<λ, where P is the particle size, and λ is the wavelength of the incident light [11,12]. As a consequence, the scattered light by the milk contains information about these particles and can be used to determine the quality of the milk. It is important to mention that most of the scattered light goes in a forward direction (Figure 1). Besides, the milk usually has a temperature around 37 °C when it leaves the udder; then, the particles are slowly suspended and the Brownian motion is avoided [10].

It is important to mention that the concentration and particle diameter made the milk a turbid and opaque medium. However, forward scatter intensity transmitted light (*I_s_*), which is the main scatter direction, can be described by [13]

(1) $$I_{s}=I_{o}e^{-\mu_{s}*d},$$

where $I_{o}$ is the intensity of the incident light, $\mu_{s}$ the scattering coefficient of the medium, and d the milk container length (the interaction length). Nevertheless, the scatter presented by protein and fat should be distinguished and considered; then, the scatter transmission ratio ($I_{STR}$) can be expressed by [13]

(2) $$I_{STR}=\frac{1}{m}e^{-\left(\left(\mu_{sf}+\mu_{sp}\right)-1\right)},$$

This ratio depends on the uniform scattering coefficient m, the scattering coefficient of fat ($\mu_{sf}$) and the scattering coefficient of protein ($\mu_{sp}$). It is important to mention that the scattering coefficient will depend on the ratio between the cross-sections of the incident light and the output light. With the purpose of considering the laser beam properties and the shape of the analyzed particles, the uniform scattering coefficient m is introduced. This coefficient depends on the cross-section area of the light source ($S_{0}$) and the surface of the particle ($S_{p}$), in the milk, we could consider the particles as spherical elements, then $m=S_{p}/S_{0}$ [13].

In raw milk, estimating the refractive index (RI) is a challenge due to the dispersion caused by fat globules (opacity), temperature and wavelength. The refractive index of cow’s milk at 20 °C is between 1.3440 and 1.3485 [5]. However, several works consider the analysis of the refractive index as an element to predict fat and protein concentration. At this point, the Cauchy’s formula will be the best model to approximate the refractive index [14]

(3) $$n\left(\lambda \right)=I+\frac{J}{\lambda^{2}}+\frac{K}{\lambda^{4}},$$

When the milk is illuminated, the absorbance is presented; this effect is governed by the Beer-Lambert’s law and it is described by [15]

(4) A=log10(IoI)=d∗ε∗c,

Here, the absorption (A) depends on the optical path length (d), molar absorptivity (*ε*), and concentration (c). The product of these elements can be estimated by the logarithm response of the ratio between the intensity incident light ($I_{o}$) and the intensity-transmitted light (I). Then, the concentration of some particles (fat and protein) can be estimated by computing the absorption.

The optical properties described above are presented every time a milk sample is illuminated by light. However, the challenges of quantifying these properties are usually a real challenge, for instance: the scattering and absorptions techniques are susceptible to power source fluctuations. In addition, both of them are sensitive to optical alignment. Moreover, scattering needs a calibration due to multiple ray trajectory. At the same time, absorbance implies the detection of lower concentrations due to the higher concentrations affect a refractive index deviation. Then, the techniques proposed need to consider their intrinsic challenges. In addition, the methods need to consider aspects such as the source light’s cost, scattering cells (dimensions and alignments), the detector and its optics (optical filters and semiconductor technology), as well as electronic challenges such as amplifiers, filters and processing time. At this point, scattering, absorption, and refraction have been used as the principle of operation of most proposed optical sensors, which are based on intensity modulation, and only a few methods employ phase modulation.

Moreover, it had been demonstrated that the wavelength of the incident light exhibits a dependence on the scattering, absorption and refraction effects; as a result, different responses can be expected through the light spectrum. McCarthy [16] in 2002 reported that when the light interacts with the milk, absorption and scattering effects are presented at the same time due to the fat globules’ dispersion, casein micelles, and proteins in the whey. For example, the riboflavin absorbs light at 470 nm causing the yellow-green color of whey; here, it was validated that the yellow color of the fat is related to the β-carotene that absorbs light at 460 nm. In the UV region, some proteins like tyrosine and tryptophan absorb light at 280 nm and emit fluorescence at 340 nm; these UV-centered wavelengths are used to estimate protein content [16]. Inside the IR region, some elements in milk present different absorption wavelengths, for instance proteins absorb light at 6465 nm, lactose at 9610 nm, and some groups of lipids at 5723 nm [16]. Fox et al. [5], 2015, point out that the milk exhibits light absorption between 200 and 380 nm; here, the authors validated this effect is related to the protein presented in the milk. Moreover, the absorption effect observed between 400 and 520 nm was related to the fat percentage in the milk.

## 3. Spectroscopy Techniques

The light–milk interactions can be studied by spectroscopy, where the light interacts with the milk’s components; hence, several phenomena could be assessed [17]. In this paper, the possibility of determining the characteristics of the milk in a minimal amount of time in comparison with laboratory analyses is presented. To discuss the several milk analysis techniques, they are separated according to the light wavelength spectra operation from UV, VIS, and IR [17].

**Figure 2:**
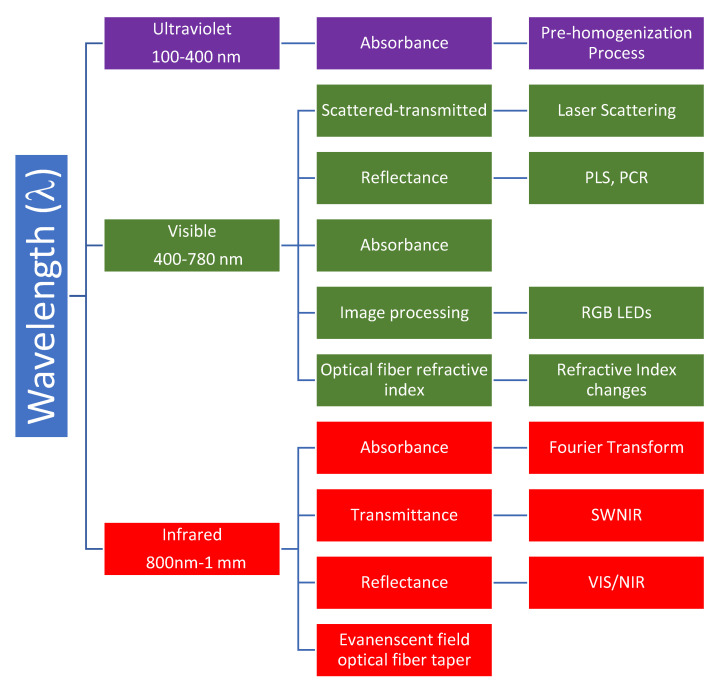
The techniques reported in the literature review by the wavelength operation.

The techniques from the studies founded in the literature review are determined in Figure 2. It can be pointed out that only the techniques focused on light–milk interaction are considered.

### 3.1. UV Techniques

The UV wavelength range is limited from 100 to 400 nm; moreover, the UV presents a sub-range such as UV-A (315–400 nm), UV-B (280–315 nm), and UV-C (200–280 nm). The range from 100 to 200 nm can be only achieved in a vacuum condition. These ranges are related to some matter properties, for instance, UV-A is used for curing materials and fluorescent labels. Meanwhile, UV-B is used for sterilization and food process. Usually, this range has a negative impact on biological samples. Finally, UV-C is related to water purification and the detection of some agents.

One of the first uses of UV light is related to the pre-homogenization process for raw cow milk [18]. Here, a mixture of reagents were used to disperse fat globules and perform a spectrophotometric method to determine fat and protein. To estimate total proteins, they measured the absorbance at 280 nm after adding 5 mL of acetic acid diluted to 97% volume to 0.05 mL of milk. For fat determination, they added a solution of 20% urea and 0.2% imidazole, measuring absorbances at 400 nm. The use of reagents eliminates the need for pre-homogenization of the samples, but there is a high risk of skin irritation manipulating them.

Kuaye, in 1994 [19], presented a method based on the use of a strong alkaline solution to change the optical response of the tyrosine; here, it was demonstrated that the absorption-centered wavelength can be shifted to wavelength values in the range between 248–256 nm. It is important to stress that, using absorbance, a linear relationship between the protein concentration and the optical response can be expected. Before the performance of the test, a filtering of the prepared samples is needed. The method measures the light absorbed for the aromatic amino acids.

Several years later, Lüthi-Peng and Puhan, in 1999 [20], developed a simple and rapid technique to determine protein and casein content in cow milk, based on spectrophotometry using the fourth derivate in the 283.5 and 294.5 nm wavelengths. Moreover, if the proposed method is compared to the Kjeldahl method [21], there is no statistical difference. However, a specialized preparation of the samples is needed, which consists of heating, homogenization, and preservation, causing the method to become time-consuming.

Forcato et al. [22], in 2005, proposed an alternative method to estimate milk fat content based on spectrophotometry in the UV range 208–215 nm. In order to precipitate proteins that could interfere with the measure, 3 mL of ethanol was added, stored for 1 h at −20 °C, then centrifuged for 15 min at 13,000 rpm. The method was compared with Milko-Scan (Foss, Denmark), pointing out that accuracy and precision were acceptable. As can be observed, a large amount of time, reagents, and specialized equipment are needed.

The spectra analysis below 550 nm lacks relevant information about the principal components of raw milk [3]; therefore, just a few UV-based methods are available. Besides a reliable alternative, UV techniques are time-consuming and preparation of samples is needed. On the other hand, it is necessary to include correction factors, and thus the scattering interference of fat globules. Although it is a rapid method, it is not reliable to implement for in-line determinations due to the chemical preparation of the samples.

### 3.2. VIS Techniques

Turbidimetric methods were used to complement laboratory analysis until, in 1959, Haugaard and Pettinati [23], presented the first photometric methods based on light scattering at 600 nm to estimate fat content. A calcium chelating agent was used to eliminate turbidity. To prevent fat globule clumping, a special solution was prepared and added to diluted milk. According to the authors, the multiple scattering phenomena is avoided, due to the layer of milk measured being very thin, and casein turbidity is eliminated.

In 2006, Xin et al. [13] provided a simple and rapid method to determine fat and protein content in liquid milk based on a laser beam at 632.8 nm. The samples were prepared by adding a mixture of chemical reagents to dilute milk beside a pre-homogenization process. In order to avoid light scattering, the dimension of the particles of milk fat globules and protein remain at 2000 and 120 nm, respectively; these dimensions agree with the scattering range limits 0.1 λ < *P* < λ [11,12]. The light scattering caused by other particles diluted in milk, such as lactose and salt, among others, can be ignored because their sizes are much less than protein and fat. This method can be used as an alternative in laboratory determinations but is difficult to install in a milking parlor.

Moreover, in 2009, Muniz et al. [24] mentioned the convenience of developing systems based on the VIS range due to the low cost of it. They also point out the benefits to add Partial Least Squares (PLS) regression or Principal Components Regression (PCR); using these statistical techniques, the final results can be improved. They studied the three phenomena: scattering, transmittance, and reflectance. The system implemented is composed of two light sources: VIS and IR. The reflected, transmitted and scattered light is captured, converted to data, and stored in a computer.

In 2012, Bogomolov et al. [25] developed a technique using a visible range; the total amount of fat and total proteins were estimated. They demonstrate that light scatters in the visible range dominate in fat and protein spectra. The absorbance spectra measured were acquired from 400 to 1000 nm, claiming that the wavelength range is suitable to be used to implement an online system. However, the experimental design of the milk samples has a short range, the variation in protein was between 2.60% and 3.20%, and the variation in fat between 3.02% and 3.98%. For that reason, the real implementation of the technique is not reliable. The prediction model use spectra values from the NIR region, for that, the method is not completely based on visible light.

A different approach was presented by Kucheryavskiy et al. in 2014 [26]: they analyze the transmitted light trough milk samples to correlate fat and total protein using image processing. Images were acquired by a conventional digital camera, and red, green, and blue (RGB) LEDs, in a dark cabin. Before the images were taken, they fixed the exposition time from the camera, giving more time for the camera sensor to capture light. The authors assure that they can correlate the amount of fat and protein in raw milk with the concentric rings generated by the image processing system. This method gives a practical and low-cost approach to the development of online systems. The authors assure that the results are closer to the spectroscopic analysis presented in 2013 [27].

Furthermore, in 2019, Gowri et al. [28] developed an optical fiber probe to estimate fat content in milk. They experimentally demonstrated that milk altered with water can be detected by analyzing the RI changes. The light absorbed is reduced with the increase in water to the milk sample. At 530 nm, the best results were achieved with a sensitivity of 0.15 ∆A/∆% fat. This technique could be a good solution in order to easily determine fat content; however, the probe must be cleaned for 3 min after each determination, and the techniques remain an invasive way to determine fat content.

### 3.3. IR Techniques

The opacity in milk is caused by the scatter created by wavelengths smaller than fat globules in the VIS region. These phenomena are not presented by NIR wavelengths, because they are larger than fat globules’ diameters. Based on that premise, Goulden [29], in 1964, presented the principles of a method to perform a quantitative analysis of fat, protein, lactose, total solids and solids nonfat of milk using IR absorption. In the year of 1993, Luinge et al. [30] proposed the use of Fourier Transform (FT) in infrared spectrometry as an alternative for the fat, protein, and lactose content determination. They reported no significant differences among laboratory analysis. According to the authors, the FT infrared spectroscopy, in combination with any of the regression methods such as PLS or PCR, is recommended for in-line analysis of fat, protein, and lactose.

In this way, Šašić and Ozaki [31], in 2001, presented a study where raw milk samples were analyzed in the 700–1100 nm range (short-wave, near-infrared, SWNIR). The authors presented the potential of the SWNIR wavelength, to perform both qualitative and quantitative analysis. PLS calibration models were used for fat, protein, and lactose predictions, however, they only obtained reliable results for fat and protein. The band at 930 nm presented the best performance for fat calibration models. For protein calibration, the wavelengths of 906, 926, 950, 864, and 1032 nm had the best results.

Taking advantage of the transmittance spectroscopy techniques, Woo et al., in 2002 [32], developed a measurement unit called MilkSpec-1 that determines fat, lactose, and protein content in a non-destructive assay. The samples of raw cow milk were non-homogenized and inserted into the system by using general glass tubes. The NIR spectrums acquired with the system are pretreated with multiplicative scatter correction (MSC) and derivatives in order to diminish the scattering effects caused by fat globules and casein micelles. They found fat content variation in the 926 nm wavelength in the MSC spectrum and absorption at 1020 nm from the MSC and second derivative spectrum, related to proteins. The results observed from the PLS calibrations show that the MSC spectrum is the best for the prediction of fat and protein content with 11 and 13 factors, respectively, and no pre-treatment with 14 factors for lactose.

Moreover, in the year 2004, Etzion et al. [33] determined protein content in raw milk based on measuring absorbance in two wavelength ranges: 5882–6666 and 9090–9433 nm. In the band around the 6097 nm, it can be found the presence of water; in order to avoid water absorption, an automatic procedure for water subtraction from the spectra was applied [34]. For the protein quantification, three data-processing methods were implemented: integration of the amide bands spectra, PLS, and principal components analysis (PCA) with artificial neural networks (ANN). The third approach (PCA with ANN), is based on the parameters from PCA, fat and lactose content as input variables, and protein prediction as a result. The authors showed that, taking into account the fat and lactose as input variables, the prediction error is reduced. Despite the low error of prediction of less than 0.5%, the ranges of protein content from the calibration samples are from 2.47 to 3.95%; for that, at the moment of implementation in a real environment, milk samples would be outside this range, and the calibration models would not be capable of performing a real estimation.

By the year 2007, Kawamura et al. [35] reported an online monitoring system to estimate fat, protein, and lactose over the 600–1050 nm wavelength range. The system collects a milk sample of 230 mL to a chamber where a sensor acquires transmittance spectra every 10 s. In order to develop calibration models, PLS was used without spectra preprocessing, such as smoothing or derivatives. The coefficient of determination and standard error of prediction (SEP) obtained for fat, protein, and lactose were r^2^ = 0.95, SEP = 0.42%; r^2^ = 0.91, SEP = 0.09%; and r^2^ = 0.94, SEP = 0.05%, respectively. The authors prove that, for an online estimation system, it is not necessary to perform homogenization and pre-treatments of the spectra acquired.

One year after this, Kawasaki et al. [36] developed a milking robot that can be used to determine milk quality in real-time. Installed in the milking parlor, the system has a milk chamber equipped with the lamp, thermocouples, cooling fan, and the optical fiber. A continuous milk flow around 230 mL is measured in transmittance mode with the spectrum sensor in the wavelength range of 600–1050 nm. The system is based on NIR spectra and PLS analysis for calibration model development. However, to maintain the precision of the system, it is mandatory to update the mathematical models periodically, converting the system to an impractical method.

In 2011, Aernouts et al. [2], investigated the best technique to estimate fat, protein, lactose, and urea content in raw milk, based on the VIS/NIR wavelength and two methods: reflectance and transmittance. The results show that reflectance spectra are accurate to estimate fat and protein content, transmittance for acceptable lactose estimations, and neither (reflectance nor transmittance), are adequate for urea content estimation. The authors assure that wavelength ranges between 400 and 1000 nm gave no information to accurately predict fat content, possibly because of the thin film used (1 mm); therefore, this method is not adequate to perform online analysis. Essentially, the experiment was conducted over NIR spectra and, for the PLS calibration models, the whole VIS range was not considered in order to get higher correlations.

A couple of months later, Muñoz-Ossa et al. [37] developed a tapered-optical-fiber-based sensor to measure fat content in fresh milk. The taper consists of a slimmed-down section of the fiber to increase sensitivity with the media. The tapered section was immersed in fresh milk with different fat concentrations. As a result, the fat micelles are crowded in the taper region and the optical power signal was attenuated. The signal decreased by a rate of around 0.00425 mW as the fat content increased. This invasive method can improve the linearity with a taper with a higher diameter, but is less sensitive.

Additionally, in 2012, Melfsen et al. [38] designed and evaluated an in-line system to predict concentrations of fat, protein, lactose, urea, and somatic cell count. The system analyzes the spectrum from 851 to 1649 nm, during the milking process. Before the milk samples from each cow were measured, a dark and white reference spectrum was acquired. The spectra were measured several times during the milking process every 500 ms from a layer with a thickness of 30 mm. For every 2 kg of milk analyzed, the spectra were averaged, and a reference milk sample was bypassed. The authors show that, during the milking process the spectra is changing, and the reflectance intensity increases as the time of milking increases. This change in the spectrum is attributed to the scattering effects caused by the particle sizes. To avoid intensity shifting, the spectra were normalized. However, this preprocessing of the spectra could dismiss important intensities that can be related to the compounds of interest. Despite the very acceptable results of the predicted versus actual concentration values from the developed system, the scattering effects are presented and reduce with pre-treatment of the spectra. According to the authors, NIR-based systems will not achieve the accuracy recommended for laboratory analysis.

In 2013, Feng et al. [39], based on non-dispersive short-wave near-infrared (NDSWNIR) spectrometry in the range from 600 to 1100 nm and PLS regressions, determined the fat, protein, and lactose of unhomogenized raw milk samples. The analyzer is composed of a single-beam optical system and can perform estimations within 1 min, where 30 s is used for heating the milk sample, and 30 s to perform the measurement. This procedure uses the absorbance from selected wavelengths according to their probable band assignment of the main components of milk, resulting in only NIR wavelengths. This approximation is suitable to implement as an off-line system to estimate the principal components of milk; however, the need to heat the samples makes the proposal time-consuming.

Bulk optical properties (BOP) of raw milk can correlate its composition (fat, protein, lactose, etc.) and physical properties (distribution of fat globule and casein micelles size) [3]. In 2015, Aernouts et al. [3] developed an optical system to measure total reflectance and total transmittance from a milk sample inside a cuvette of 600 µm of path length, composed of a supercontinuum laser (500–2250 nm) with a monochromator. They found a correlation (r ≤ 0.762) between fat content and the bulk absorption coefficient (${µ}_{q}$) in the NIR wavelengths, and a high correlation (r ≥ 0.975) between fat and the bulk-scattering coefficient (${µ}_{s}$) in the 1300–1400 nm range. This is because the size of the fat globules has the slightest effect on ${µ}_{s}$ in that wavelength range, influenced essentially by the fat content itself. Despite the low correlation coefficients, the study of the BOP can provide information in order to test different arrangements of the sensing system, and how the interaction between the light source and turbid media (in this case, raw milk) occurs.

With the use of a W-type optical fiber sensor system, Zhu et al. [40] developed a rapid method to measure fat content in raw milk. The system was evaluated at different milk temperatures, finding that 40 °C was optimal to perform the test. The system is equipped with a light source with wavelength centered at 1060 nm; this beam is divided and travels into a W-type fiber. One beam interacts with the surface of the milk sample, and the second beam interacts with distilled water; then, the reflected lights are captured by two detectors. The authors show this system exhibits an error range of ±3%; however, the range of the reference fat content used with the system is very limited, around 0.17% and 0.23%. In order to establish the best temperature of the raw milk to estimate fat content with the proposed system, different temperatures were tested; despite the mentioned optimal temperature, no significant difference was presented between the treatments (25, 30, 35, 40 and 45 °C). This approach can be used as a rapid method to estimate fat content; nevertheless, the significantly different fat concentration must be evaluated in order to develop a practical system.

With the support of PLS analysis, Niero et al. [41] investigated the capability of the mid-infrared region to predict the protein content of bovine milk. The samples were preserved with a mixture and stored at 4 °C. The spectra from the 110 milk samples were acquired with the MilkoScan FT6000 (Foss Electric A/S, Hillerød, Denmark), and the absorbance was calculated and uninformative variable elimination and PLS were used to create calibration models, eliminating the wavelengths where the water absorbs light. The authors report that the best prediction model for total protein is an $R^{2}=0.88$. Despite the proposal being useful for practical approaches, the method still remains as an off-line method of analysis. The construction of predictive models with PLS has been reported several times; however, a lot of statistical compute effort must be used, as well as calibration, in order to predict efficiently.

Ragni et al. [1], in 2016, presented an inexpensive and easy-to-implement system based on the light that emerges from a material detected by a characterized photodiode working in the NIR range. A tungsten lamp varies its intensity by a digital signal as a reference, and the photodiode captures the transmitted light through the milk sample. The authors attribute a strong influence of fat content to the light-scattering effects, in the article, it can be observed that the best region is from 800 to 900 nm; here, a good correlation between fat content and scatter light was achieved. As the milk fat content increases, the irradiance detected by the photodetector is lower. An ANN was added to the system, reporting the prediction with an $R^{2}=0.992$. The proposed technique can be implemented in both online and offline systems; however, the effect of the temperature on the system seems to be the main problem. On the other hand, the amplitude modulation used in the article can vary as the lamp gets older; for that reason, multiple calibrations must be performed constantly, to the point of necessary lamp replacement.

In the same year, Li et al. [42] presented a rapid estimation of fat and protein content in raw milk, applying different techniques as spectral denoising to eliminate the effect of solid particles, surface scattering, and the optical path difference; derivative denoising in order to eliminate the baseline and overcome the overlap of the spectral bands; genetic algorithms; and PLS to construct a calibration set from the selected samples. The validation test shows, for protein, an $R^{2}=0.8010$ and, for fat content, an $R^{2}=0.9100$. Despite the results, the range of the composition of the protein and fat samples was short (2.82–3.39 and 3.28–4.18, correspondingly), and the wavelengths where they perform the test were the MIDIR from 2564–25,000 nm, where the spectrum is affected by the presence of water and other components of the raw milk. The milk samples were not homogenized, and the scattering effects were not taken into consideration. Further, as mentioned before in this review, the amplitude modulation can provide relative errors with the passage of the time, due to the aging of the illumination system. This method can be implemented in online processes, but the optical spectrum analyzer is going be expensive.

Biological factors such as mastitis, lactation, feeding, season, breed, among others, could cause systematic errors in determinations with IR methods. For example, carboxylic acids formed on the fermentation of lactose cause interference absorption in the wavelengths that protein is measured by [42]. For this reason, calibrations of the IR systems are needed constantly. In NIR-based systems, calibration must be performed before any measurement with representative samples and another set to validation, besides the use of multiple linear regressions (MLR), PCR or PLS [43].

## 4. Challenges and Prospects

Throughout the years, different approaches have been developed in order to create new online systems to estimate fat and protein content in raw milk. However, to date, there is a gap in the technological development of techniques that can be implemented as a robust, precise, and cheap system. Several opportunity areas for addressing the challenges are as follows.

### 4.1. Costs

The NMR, as well as the IR technologies, has been declared as a highly expensive to be implemented in a dedicated system [34]. The necessary instrumentation to implement a NIR prototype requires a specialized light source and a wavelength detector. Once the signal responses are acquired, they must be processed in sophisticated software. These three parts of the systems could represent 70% of the cost, and could only be used as offline analysis, and implemented only for one workstation. If a system is constructed with these elements per milking station, the prize could be more than the whole milk production system. The specialists in the development of photodetectors must improve their products to achieve a similar response for the detectors from the optical spectrum analyzers.

### 4.2. Embedded and Integration

Once the techniques are developed and the materials are proposed, it is time to implement the mathematical models into an embedded system. Here, we detect a gap and an opportunity for the development of embedded systems, where the signal could be processed in real-time. Technological approaches like microcontrollers, microprocessors, and field-programmable gate arrays (FPGA), could be the solution. Several attempts to develop embedded sensing systems based on microcontrollers can be observed for detecting water alteration in milk samples [44], such as a telemetry system for an amperometric biosensor application [45], a monitoring system for greenhouses using microprocessors [46], fast image processing based on FPGA [47], to reference some applications.

In this appreciation, embedded systems endow the production system as a precision system controlled by autonomous subsystems, which will be collecting data continuously, with the possibility of self-identification of individuals and exporting information in real-time; in this way, the producers could make decisions more adequately, with the precise information available all the time.

In order to have the technologies incorporated in the milking parlors, there must be an update in the development of systems with low power consumption, so that the systems can operate for prolonged periods with less energy. The system must be the least invasive system that is possible inside the milking parlor; furthermore, the materials used for the construction must support mechanical stresses, high relative humidity, and not interfere with the cow’s path, whilst being assembled as a robust integrated system.

### 4.3. Self-Calibrated Mathematical Models

Most of the techniques reviewed in this paper must be recalibrated with the passage of the time. This activity must be eliminated in order to have a complete system installed in milking parlors. The development of evolutionary algorithms, and artificial intelligence like machine learning, ANN, fuzzy logic and others, could be added to the analysis system. These techniques could help to make the process of adjustment of the mathematical models less time-consuming, and may be transparent for the final user. Even though the cost of implementation into an embedded system could be high, there is an opportunity for the developers to integrate these techniques into a low-cost platform.

The requirements for the good development of cows in lactation, pregnancy, and growth states are crucial to the production systems. There are mathematical models to help the producers make decisions; for example, to estimate cattle requirements for nutrients supplies following different production circumstances [48]. These models could be more efficient if they were auto-adjustable with time, and had smart systems to feed them with the required data.

### 4.4. Communication

Technological developments in the area of communications can provide a great opportunity for production systems to increase operating efficiency, prevent diseases promptly, and maintain the constant and efficient monitoring of milk quality [48].

It is more common that every technological system has wireless communication between the sensing system and a database. In this area of knowledge, a milk analysis system with the internet of things (IoT) included, could be attractive to milk producers. Here, the development of communication arrangements, wireless protocols, and dynamics databases, where the information can be accessed from any access to the internet from worldwide could be desirable. The analysis system could have a permanent connection between the recently analyzed cow and the producer.

IoT technologies have been tested to assist producers in order to increase production by managing the cattle. Individually, the automatic system can determine the required feeding and milk production [48].

Technologies like radio frequency identification could be convenient, using radio tags to identify each milking system onto a wireless network. IoT integration could bring to the system the potential to increase productivity, as well as the reach of new markets, self-organized systems, and auto-collect data from each node of the net [47]. Nowadays, IoT brings data security and cloud-computing applications enabling the creation of smart systems in real-time, supports a large number of users, and the system still can be operated in both wired and wireless environments [47].

## 5. Trends

**Figure 3:**
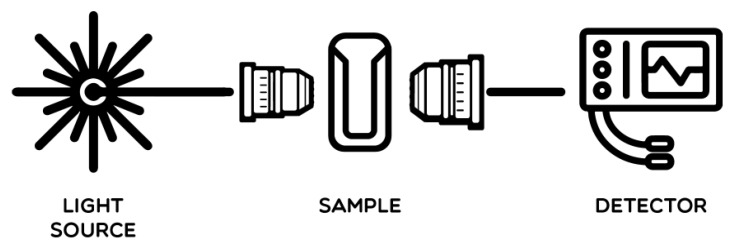
General diagram of the spectrophotometric methods.

After reviewing all the techniques that have been using throughout the years, we can point out the spectroscopy as an interesting method for non-invasive estimations by combining rapid predictions, at low cost, and the opportunity to develop in-line applications. The use of systems based on spectroscopy techniques has increased in the past years. In the spectrophotometric methods, the main components are the light source, the sample, and the detector (Figure 3). The light source may vary from a tungsten lamp, laser, light-emitting diode (LED), or a supercontinuum source; the generated light travels through an optical fiber until the detector system that could be an optical spectrum analyzer, thermopile or photodetector. The sample to analyze is deposited in a container; usually, quartz cells with 1 mm light path are used. In particular cases, it could be necessary to use coupling devices that modify light properties such as attenuators and lenses [49]. This last point is where a broad configuration could be used in order to bring out specific compounds from the samples. A special arrangement is the interferometers, which are optical devices that separate and then recombine the light beam.

When the interferometer is integrated onto an optical system, it has been proved that the sensitivity increase [50], besides the immunity to electromagnetic interferences, and operation in a wide range of environments. There are four types of interferometers: Fabry-Perot [50], Mach-Zehnder [51], Michelson [52], and Sagnac [53].

This technique explores the light-milk interaction when the light crosses the components inside the raw milk; however, multiple scattering effects are presented in turbid liquids like milk [10].

In order to improve the existing methods, our team is developing a new spectroscopic technique to determine fat and protein content in raw milk, taking the scattering effects into account. The method is based on the principle of light reflection through a fiber optic interferometer [54]. This optical fiber sensor is composed of a dual-response Fabry-Perot interferometer (FPI) with multimodal optical fiber; moreover, the technique detects wavelength-shifting at different regions for both protein and fat content. This new method can be implemented in online systems, resulting in its being cheap and easy to develop. The authors propose the use of one LED and a photodetector in order to avoid the construction of a multispectral analyzer. With that, a rapid, cheap, non-invasive, and in situ fat and protein determination can be implemented in milking parlors, preventing illness such as mastitis and ketosis, besides the precise estimation of the principal contents in raw milk.

In advance, the technique was tested for temperature noise variations, and because the method is based on phase modulation, temperature variations, both internal and external, show no effect on the determination of the spectra.

**Figure 4:**
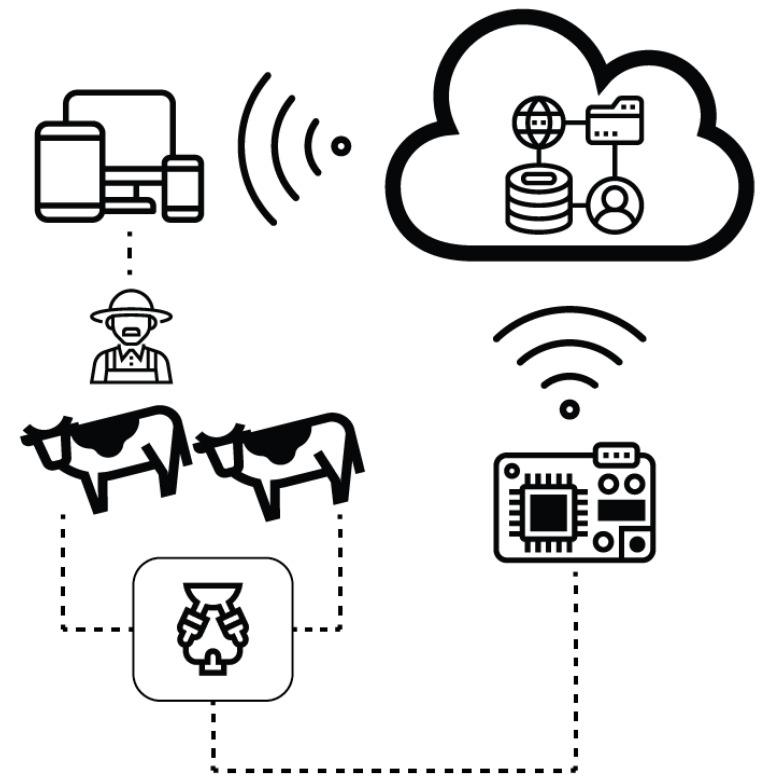
Smart farming applied to milking parlors.

In this way, our team is proposing to focus development towards interferometry optical sensors as a line of technological development. Based on the principle of developing simpler systems, a lot of development must be performed in order to have a specialized apparatus. Here, we can refer to the challenges mentioned before in this review. New trends in every production area are about smart farming [55], where greenhouses could improve production by optimizing grow parameters, fish farms with recirculation, and real-time analysis of the fish behavior to establish feeding parameters, and, using the same trend, implement smart milking parlors. The system (Figure 4) could be provided by a sensor net in order to get principal milk quality parameters with an in-line system, automatically recognizing analysis from each cow. Through an embedded system, the collected information could be processed and uploaded onto an online cloud computing platform, where smart devices such as smartphones or computers display a human–machine-interface; then, the farmers could make decisions about production, adjusting feeding parameters for each cow or the complete herd, monitor health, or prevent illnesses. All of these production importance parameters could be suggested by the artificial intelligence that the system could learn through time.

The collaboration networks could be pointed to join efforts in order to design cheaper prototypes for well-integrated embedded systems, bringing the possibility of producing higher reachability of technology, leading to a high-integrated system, and not cut the possible applications. On the other hand, the research teams that are working on communications could contribute to adding wireless modules such as IoT protocols and technologies for the 4.0 industries.

## 6. Conclusions

It can be observed that the current trend is focusing on the development of spectroscopy techniques, in order to obtain online systems to predict milk composition in situ, without the use of chemical reagents, and in a fraction of the time compared to laboratory tests.

The potential of spectroscopy techniques widely used today has been demonstrated with the potential use of on-line systems, but still remains expensive. The best techniques are NMR, but this could only be used as an offline technique because of its cost [56].

In Table 1, the most remarkable techniques founded in the literature review to determine the principal components in raw cow milk, such as fat, protein and lactose, are summarized. Those methods are presented by reported year, and the optical characteristic used in the development. We can observe that most-used spectral range is based on the NIR wavelengths. The use of predictive models such as PLS is recurrent among authors; however, several papers report that the use of ANN and/or the use of an FTIR spectroscopy are recommended to improve their estimations [1,33,57]. However, in order to get an automatic system, neither of those articles provide an easy-to-implement, low-cost, and non-invasive system. Although the techniques reported to date work on the modeling amplitude from the spectral signal recorded, as mentioned before, this method involves several limitations. For this, a new interesting proposal could be converting the spectral signal to the time-frequency domain, avoiding those limitations.

A recent non-invasive technique is introduced, presenting promising results based on the use of optical dual response FPI, VIS light reflection, and phase modulation, taking the multiple scattering effects into consideration in order to perform rapid estimations of fat and protein rapid.

The recent advances presented in the literature results show a valuable effort to be closer to the selection of the best technique for rapid and precise estimation and, consequently, the development of online systems.

## Figures and Tables

**Table 1:** Principal methods to estimate main components in milk based on optical properties.

Year	Acquisition Method	Component Determination	Spectral Range (nm)	Data Processing	Validation			References
					R2	Sensitivity	RMSEP (%)	
2019	Reflection	Fat	500		0.9763	616 pm/% fat		[54]
		Protein			0.878			
2019	Absorbance	Fat	530			0.15 ∆A/∆% fat		[28]
2016	Transmission	Fat	2500–25,000	PLS	0.91007989		0.045329	[42]
		Protein			0.8010929		0.0207505	
2016	Scatter	Fat	400–1000	PLS			0.05	[41]
		Protein					0.03	
2015	Scatter	Fat	1300–1400		0.975			[3]
2014	Transmission	Fat	400–700	PLS	0.973			[26]
		Protein			0.974			
2013	Scatter	Fat	400–1100	PLS	0.952		0.13	[27]
		Protein			0.959		0.04	
2013	Transmission	Protein	600–1100	PLS	0.932		0.201	[39]
		Fat			0.981		0.172	
		Lactose			0.933		0.247	
2012	Transmission	Fat	400–1000	PLS	0.915		0.05	[25]
		Protein			0.964		0.03	
2011	Reflectance	Fat	1000–1700	PLS	0.997		0.047	[2]
	Transmission	Protein	400–1700		0.90		0.162	
	Transmission	Lactose	400–1700		0.883		0.115	
2008	Transmission	Fat	600–1050	PLS	0.95		0.25	[36]
		Lactose			0.83		0.26	
		Protein			0.72		0.15	
2007	Transmission	Fat	600–1050		0.95		0.42	[35]
		Protein			0.91		0.09	
		Lactose			0.94		0.05	
2004	Reflectance	Protein	5800–9400	PLS			0.22	[33]
2002	Transmission	Fat	700–1100	PLS	0.999		0.06	[32]
		Lactose			0.964		0.10	
		Protein			0.97		0.10	
2001	Transmission	Protein	800–1100	PLS	0.996		0.087	[31]
